# Effects of HAT-CN Layer Thickness on Molecular Orientation and Energy-Level Alignment with ZnPc

**DOI:** 10.3390/molecules28093821

**Published:** 2023-04-29

**Authors:** Eunah Joo, Jin Woo Hur, Joon Young Ko, Tae Gyun Kim, Jung Yeon Hwang, Kevin E. Smith, Hyunbok Lee, Sang Wan Cho

**Affiliations:** 1 Department of Physics and Engineering Physics, Yonsei University, 1 Yonseidae-gil, Wonju-si 26493, Republic of Korea; 2 Department of Physics, Boston University, 590 Commonwealth Avenue, Boston, MA 02215, USA; 3 Department of Physics and Institute of Quantum Convergence Technology, Kangwon National University, 1 Gangwondaehak-gil, Chuncheon-si 24341, Republic of Korea

**Keywords:** HAT-CN, ZnPc, UPS, XAS, energy-level alignment

## Abstract

Efficient energy-level alignment is crucial for achieving high performance in organic electronic devices. Because the electronic structure of an organic semiconductor is significantly influenced by its molecular orientation, comprehensively understanding the molecular orientation and electronic structure of the organic layer is essential. In this study, we investigated the interface between a 1,4,5,8,9,11-hexaazatriphenylene hexacarbonitrile (HAT-CN) hole injection layer and a zinc-phthalocyanine (ZnPc) p-type organic semiconductor. To determine the energy-level alignment and molecular orientation, we conducted in situ ultraviolet and X-ray photoelectron spectroscopies, as well as angle-resolved X-ray absorption spectroscopy. We found that the HAT-CN molecules were oriented relatively face-on (40°) in the thin (5 nm) layer, whereas they were oriented relatively edge-on (62°) in the thick (100 nm) layer. By contrast, ZnPc orientation was not significantly altered by the underlying HAT-CN orientation. The highest occupied molecular orbital (HOMO) level of ZnPc was closer to the Fermi level on the 100 nm thick HAT-CN layer than on the 5 nm thick HAT-CN layer because of the higher work function. Consequently, a considerably low energy gap between the lowest unoccupied molecular orbital level of HAT-CN and the HOMO level of ZnPc was formed in the 100 nm thick HAT-CN case. This may improve the hole injection ability of the anode system, which can be utilized in various electronic devices.

## 1. Introduction

Organic semiconductors are a promising class of materials for flexible electronic devices such as light-emitting diodes, solar cells, and field-effect transistors [1,2,3,4,5,6]. Because organic semiconductors do not possess sufficient intrinsic charge carriers to operate devices, charge carriers must be injected from the electrodes. Therefore, achieving efficient energy-level alignment in organic electronic devices is necessary for obtaining high performance [7,8,9,10,11]. One fundamental requirement for reducing the energy barrier between the Fermi level of the anode and the highest occupied molecular orbital (HOMO) level of the organic layer is a hole injection layer (HIL) with a high work function. 1,4,5,8,9,11-Hexaazatriphenylene hexacarbonitrile (HAT-CN) and MoO_3_ are well-known and efficient HILs with the high work functions required to elevate the HOMO level of an adjacent p-type organic semiconductor [12,13,14,15,16]. Furthermore, the lowest unoccupied molecular orbital (LUMO) of HAT-CN and the conduction band minimum of MoO_3_ are close to the Fermi level of the anode, resulting in effective hole injection by withdrawing electrons from the p-type organic layer. This phenomenon is known as the charge generation mechanism [17,18,19,20,21,22].

When considering a charge-generating HIL, HAT-CN may be more efficient than MoO_3_ because it can be deposited at lower temperatures despite their similar electronic structures. However, when using an organic HIL, it is essential to consider that the electronic structure will differ according to the molecular orientation [23,24,25], as is the case for the work function of HAT-CN [26]. Furthermore, the molecular orientation of the outmost layer affects the photoemission spectra from the valence band and HOMO in the molecule [27,28]. This indicates that molecular orientation plays a crucial role, not only in charge transport via π-orbital stacking, but also in energy-level alignment. Furthermore, the altered molecular orientation and resultant electronic structure can subsequently affect the structure of the adjacent organic layer. Therefore, understanding the molecular orientation and electronic structure of organic molecules is critical for building efficient device structures. Due to the highly anisotropic nature of the π-conjugated molecules, the molecular orientation of organic thin films can significantly affect aspects of device performance, such as light absorption and charge-carrier transport [29]. 

**Figure 1:**
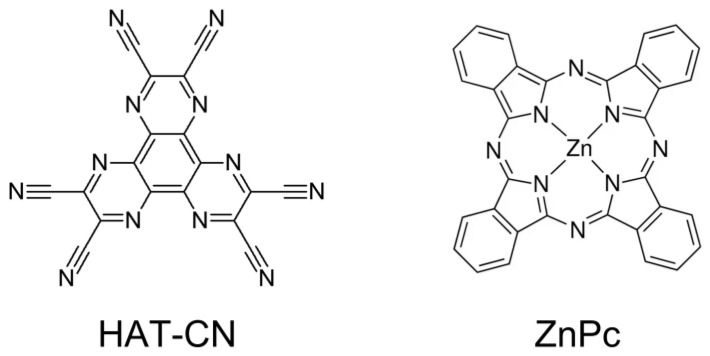
Chemical structures of HAT-CN and ZnPc.

In this regard, to further investigate the impact of molecular orientation on energy-level alignment in organic electronic devices, we conducted a study in which we controlled the thickness of a HAT-CN layer (5 nm and 100 nm) and examined its effect on the molecular orientation of a layer of zinc phthalocyanine (ZnPc), a representative p-type organic semiconductor [30]. ZnPc is widely used in optoelectronic devices, including field-effect transistors and solar cells [31,32,33,34,35,36]. The optoelectronic properties of planar ZnPc molecules are significantly affected by their molecular orientation. Therefore, the HAT-CN/ZnPc can be an interesting HIL/p-type organic semiconductor system with potential utilization in various devices. The chemical structures of HAT-CN and ZnPc are shown in Figure 1. We utilized in situ ultraviolet photoelectron spectroscopy (UPS) and X-ray photoelectron spectroscopy (XPS) to explore the energy-level alignments of the HAT-CN/ZnPc interfaces; moreover, we conducted angle-resolved X-ray absorption spectroscopy (ARXAS) to determine the molecular orientations. To understand band bending between HAT-CN and ZnPc, it may be useful to measure both HAT-CN/ZnPc and ZnPc/HAT-CN interfaces by changing the deposition sequence. However, in a previous report, the HOMO offset between 4,4′-N,N’-dicarbazolyl-biphenyl (CBP) and (4,4′,4”-tris [3-methyl-phenyl(phenyl)amino]-triphenylamine) (m-MTDATA) organic materials was found to change from −0.3 eV to 1.0 eV by the deposition sequence to due to the Fermi level pinning [37]. Therefore, we only focused on the ITO (electrode)/HAT-CN (HIL)/ZnPc (p-type organic semiconductor) interfaces, which can be actually used in optoelectronic devices. The obtained results are discussed in terms of their impact on device performance.

## 2. Results and Discussion

**Figure 2:**
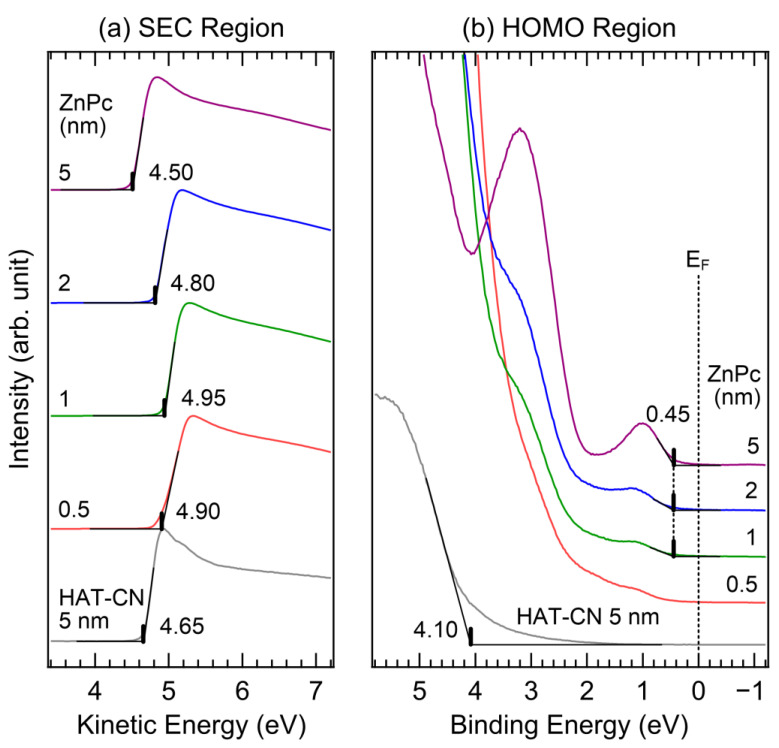
UPS spectra of (**a**) SEC and (**b**) HOMO regions of the HAT-CN (5 nm)/ZnPc (0.5, 1, 2, and 5 nm) interface.

We conducted in situ UPS and XPS measurements to investigate the interfacial electronic structure. Figure 2 shows the UPS spectra of the (a) secondary electron cutoff (SEC) and (b) HOMO regions of HAT-CN (5 nm) and ZnPc (0.5, 1, 2, and 5 nm). The SEC spectra were normalized to clearly show their shifts, and a kinetic energy scale was used to directly indicate the work function as its onset. The work function was evaluated from a UPS spectrum by subtracting the SEC position in the binding energy scale from the photon energy (21.22 eV) of the UV source. The Shirley-type background was removed from the obtained HOMO-region spectrum to accurately determine the HOMO onset. As shown in Figure 2a, the work function of the 5 nm thick HAT-CN layer is 4.65 eV, which is smaller than that from a previously reported study [38]. When the ZnPc layer was deposited at a thickness of 1 nm, the work function increased. At thicknesses of 0.5 nm and 1 nm, the work functions were 4.90 eV and 4.95 eV, respectively. However, when thickness increased, the work function decreased. At thicknesses of 2 nm and 5 nm, the work functions were 4.80 eV and 4.50 eV, respectively. These changes in the work function, from increasing to decreasing with respect to the film thickness, might be attributable to factors such as charge transfer, interface states, dipole orientation, and other related phenomena [39,40,41]. However, the exact origin cannot be resolved at present, and further research is required. In Figure 2b, the HOMO onset of the 5 nm thick HAT-CN layer is observed at 4.10 eV. Because of its low thickness, the O 2p valence band signal of the indium tin oxide (ITO) substrate overlapped. The HOMO onset of the 1 nm thick ZnPc layer was observed at 0.45 eV. As the thickness of the ZnPc layer increased, the HOMO intensity gradually increased. However, the onset position was not changed. At a thickness of 5 nm, the HOMO onset of ZnPc was observed at 0.45 eV, indicating no band bending. Unlike the SEC, the HOMO level did not change with film thickness. Therefore, the fluctuation in the work function did not significantly affect the hole-transport ability. The HOMO−1 peak was also clearly observed at 3.2 eV. The HOMO of ZnPc is composed of semiconducting macrocyclic ligands, which play a role in efficient charge transport [42].

**Figure 3:**
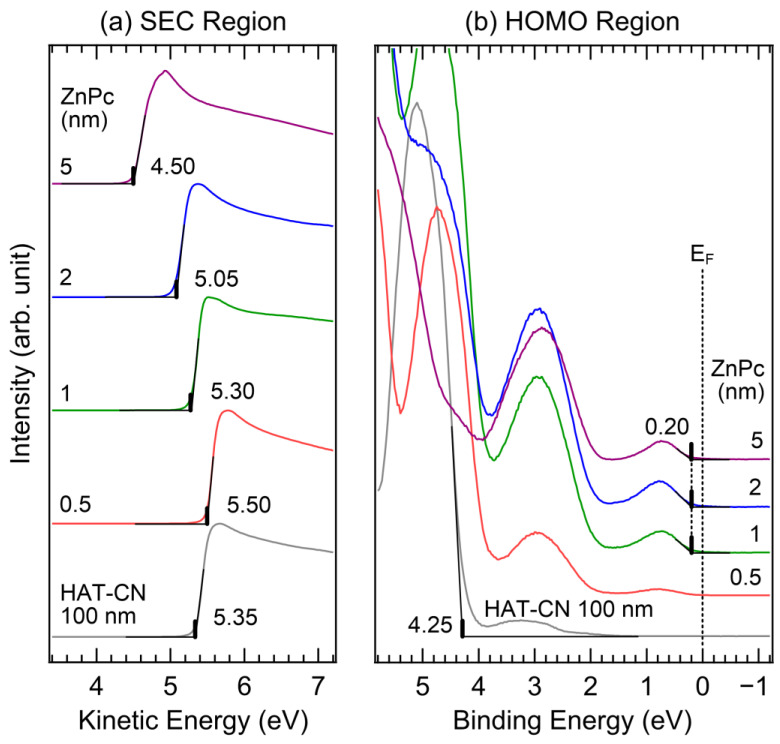
UPS spectra of (**a**) SEC and (**b**) HOMO regions of the HAT-CN (100 nm)/ZnPc (0.5, 1, 2, and 5 nm) interface.

Figure 3 shows the UPS spectra of the (a) SEC and (b) HOMO regions of HAT-CN (100 nm) and ZnPc (0.5, 1, 2, and 5 nm). As shown in Figure 3a, the work function of the 100 nm thick HAT-CN layer is 5.35 eV, which agrees well with the previously reported study due to the HAT-CN thickness being thick enough [38]. This value is 0.70 eV higher than that of the 5 nm thick HAT-CN layer. Upon depositing a 0.5 nm thick ZnPc layer, the work function increased by 0.15 eV. However, as the thickness of the ZnPc layer increased, the work function gradually decreased, similar to the case of the HAT-CN (5 nm)/ZnPc interface. At thicknesses of 1, 2, and 5 nm, the work functions were 5.30, 5.05, and 4.50 eV, respectively. In Figure 3b, the HOMO onset of the 100 nm thick HAT-CN layer is 4.25 eV. The ITO-substrate valence band features are not observed because the film thickness exceeds the UPS probing depth. The peak at 3.2 eV, which is 1.9 eV away from the HOMO peak at 5.1 eV, was caused by He I_β_ excitation and should not be considered when evaluating the charge transport levels. Upon deposition of a 1 nm thick ZnPc layer, the HOMO onset was observed at 0.20 eV. Similar to the HAT-CN 5 nm case, the HOMO onset did not shift as the thickness of the ZnPc layer increased. The HOMO onset of ZnPc was observed at 0.20 eV when its thickness was 5 nm; at this thickness, the changes in the HOMO intensity reached saturation. The HOMO−1 peak was observed at 2.9 eV. Compared to those of the 5 nm thick HAT-CN layer, the HOMO and HOMO−1 peaks of the 100 nm thick HAT-CN layer show lower binding energy positions, indicating the impact of the higher work function of the substrate.

**Figure 4:**
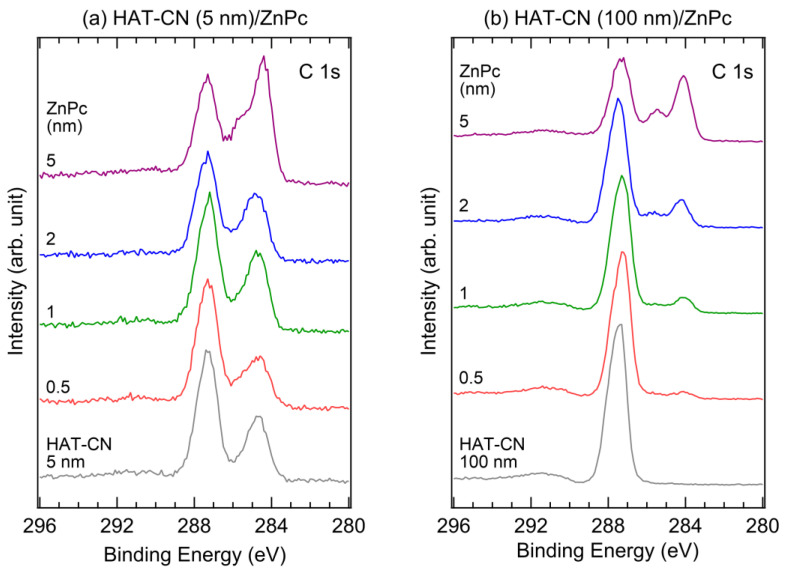
XPS C 1s spectra of an (**a**) HAT-CN (5 nm)/ZnPc interface and an (**b**) HAT-CN (100 nm)/ZnPc interface.

Figure 4 shows the XPS C 1s core-level spectra of an (a) HAT-CN (5 nm)/ZnPc interface and an (b) HAT-CN (100 nm)/ZnPc interface. In Figure 4a, two peaks are detected at 284.7 eV and 287.3 eV for 5 nm thick HAT-CN. The lower binding energy peak originated from carbon contamination in the underlying ITO substrate because of its thinness, whereas the higher binding energy peak arose from HAT-CN. The HAT-CN peak is asymmetric, owing to the two different chemical bonds of C–N and C≡N [38]. Two characteristic peaks of phthalocyanines emerged with the deposition of the ZnPc layer [43,44,45]. The peaks at 284.4 eV and 285.8 eV were assigned to the C–C and C–N bonds, respectively. During ZnPc layer deposition, both the HAT-CN and ZnPc peaks were not shifted, consistent with the HOMO level shift observed in the UPS spectra. As shown in Figure 4b, only one peak is detected at 287.4 eV for 100 nm thick HAT-CN, whereas the contaminant peak is absent because the HAT-CN thickness exceeds the XPS probing depth. After depositing the ZnPc layer, phthalocyanine peaks appear at 284.1 eV and 285.5 eV, with a spectral shape similar to that of the 5 nm thick HAT-CN sample. The lower binding energy positions of the ZnPc C 1s peaks on the 100 nm thick layer of HAT-CN were attributed to the increased work function and were consistent with the HOMO level difference. All electronic levels change in the same way upon charge transfer between two materials. However, there might be a slight magnitude difference in the energy level shifts between the UPS and XPS spectra due to their different probing depths (<1 nm for UPS and <10 nm for XPS). Nevertheless, we did not observe any XPS peak shifts during ZnPc deposition, indicating that the band bending was incidental.

**Figure 5:**
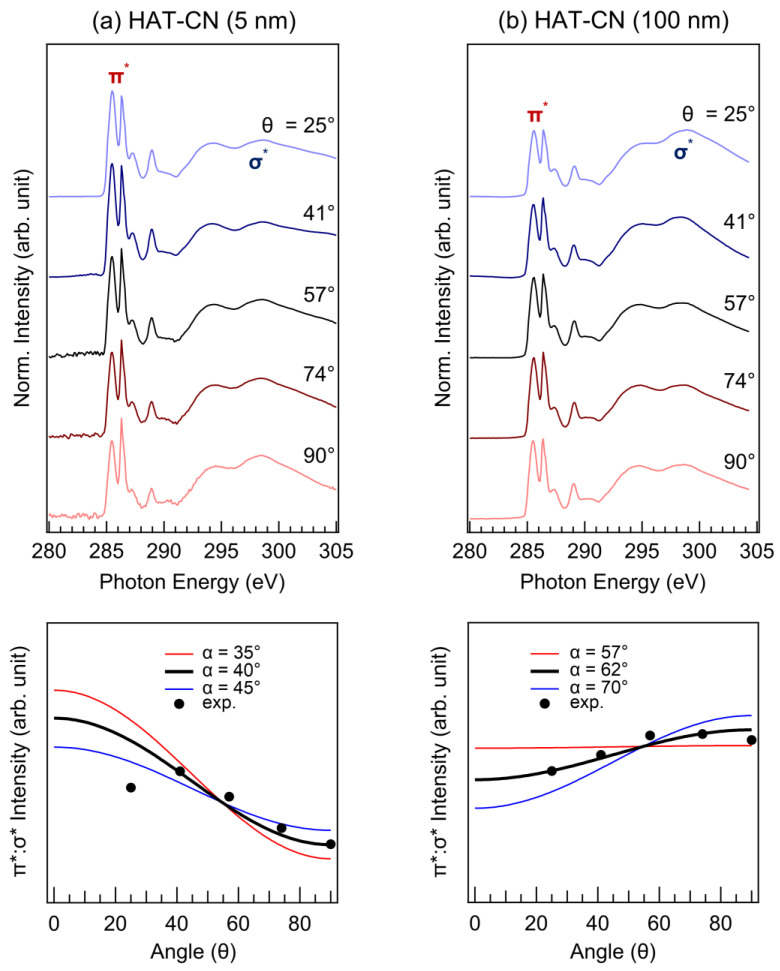
ARXAS C K-edge spectra and the relative intensity of the π*:σ* peaks of (**a**) HAT-CN (5 nm) and (**b**) HAT-CN (100 nm).

ARXAS was performed to investigate the molecular orientations of HAT-CN and ZnPc. Initially, we measured the molecular orientation of the HAT-CN layer with various thicknesses (0.5, 1, 2, 5, 15, 50, and 100 nm) using ARXAS. However, the X-ray absorption spectroscopy (XAS) intensity measured at films thinner than 5 nm was too low as the film thickness is smaller than the inelastic mean free path length for electrons at these energies. Therefore, we used films thicker than 2 nm to investigate the HAT-CN molecular orientation. Among them, we focused on the 5 nm and 100 nm thick HAT-CN films, which exhibited the most significant differences. The top panel of Figure 5a shows the angular dependence of the C K-edge spectra of the 5 nm thick HAT-CN layer (θ = 25°, 41°, 57°, 74°, and 90°; where θ is the angle between the substrate plane and photon incidence). These measurements allowed us to determine the azimuthally averaged tilts of the molecules with respect to the substrate. The resonant transitions from a C 1s to a π* orbital (photon energy range of 283–292 eV) showed a clear angular dependence, with the maximum π* intensity observed at grazing incidence. The XAS spectra were measured at the C K-edge rather than the N K-edge because the XAS intensity of the N K-edge is much smaller than that of the C K-edge. The tilt angle of the HAT-CN molecular plane (α) can be evaluated using the following relation.

$$I\left(\theta \right)\propto 1+\frac{1}{2}\left(3cos^{2}\theta -1\right)\left(3cos^{2}\alpha -1\right),\;$$

where *I(θ)* is the π* spectral intensity [46,47,48,49,50,51]. The variation in the relative intensity of the π*:σ* peaks was calculated (bottom panel of Figure 5a). The red, black, and blue lines represent the calculated ratios for α values of 35°, 40°, and 45°, respectively. The measured ratios are represented by black circles. By comparing these relative peak intensities with the calculated variations for a range of tilt angles, we determined that the HAT-CN molecules, in a 5 nm thick layer, have an azimuthally averaged tilt of 40° with respect to the substrate. Similarly, we analyzed the angular dependence of the C K-edge spectra of the 100 nm thick HAT-CN layer (Figure 5b). In this case, the resonant transitions from a C 1s to a π* orbital showed a clear angular dependence, but the maximum π* intensity was observed at normal incidence. Consequently, we determined that the HAT-CN molecules in a 100 nm layer had an azimuthally averaged tilt of 62° with respect to the substrate. These results clearly indicate that the molecular orientation of the HAT-CN layer is significantly affected by its thickness. The performance of organic electronic devices is significantly impacted by the molecular orientation of organic thin films deposited on either inorganic or organic substrates [52,53]. Numerous research endeavors have been dedicated to comprehending and subsequently regulating the molecular orientation of organic thin films on various substrates. Empirical evidence indicates that the molecule–substrate interfacial interactions or the electronic structures of substrate surfaces predominantly dictate molecular orientation [54,55]. Therefore, the molecular orientation of a 5 nm HAT-CN film is primarily impacted by the ITO substrate. In contrast, the molecular orientation of a 100 nm HAT-CN film is influenced by the HAT-CN layer underlying on it.

**Figure 6:**
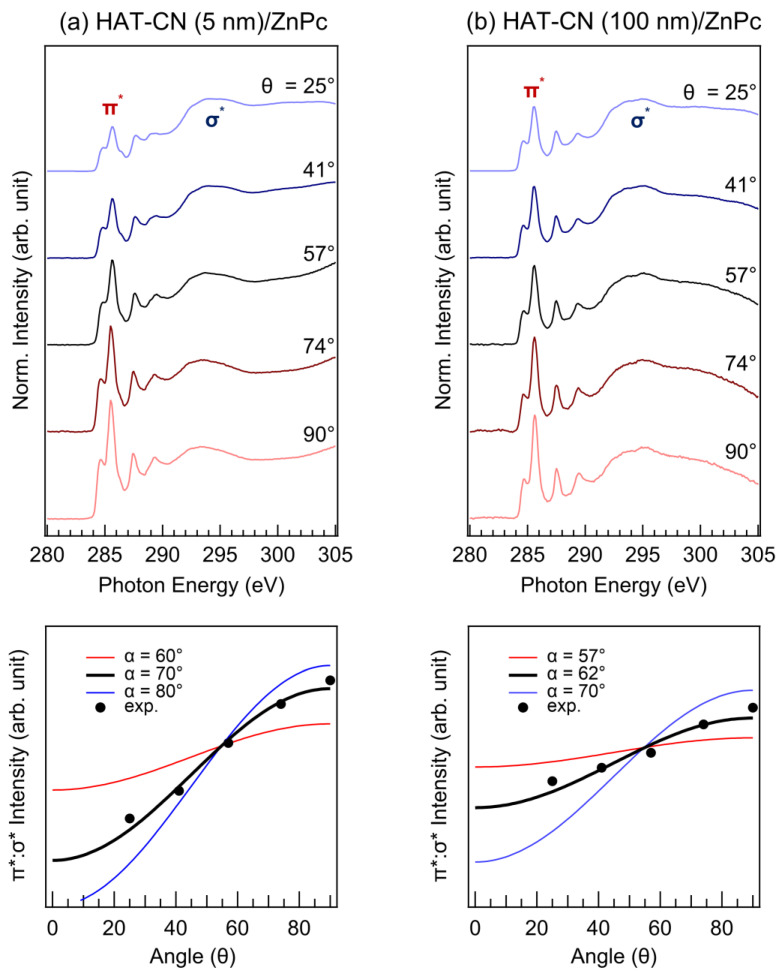
ARXAS C K-edge spectra and the relative intensity of the π*:σ* peaks of an (**a**) HAT-CN (5 nm)/ZnPc interface and an (**b**) HAT-CN (100 nm)/ZnPc interface.

In order to study the ZnPc molecular orientation depending on the molecular orientation of organic substrates, the molecular orientations of a ZnPc layer on HAT-CN layers of different thicknesses were investigated using the same method. Figure 6 shows the ARXAS C K-edge spectra of an HAT-CN (5 nm)/ZnPc interface and an HAT-CN (100 nm)/ZnPc interface (where θ = 25°, 41°, 57°, 74°, and 90°). In Figure 6a, a clear angular dependence of the resonant transitions from a C 1s to a π* orbital is observed with the maximum π* intensity observed at normal incidence. Based on this result, we concluded that the ZnPc molecules on the 5 nm thick HAT-CN layer have an azimuthally averaged tilt of 70° with respect to the substrate. In addition, we obtained the ARXAS C K-edge spectra and evaluated the relative π*:σ* intensity for ZnPc on the 100 nm thick HAT-CN layer (Figure 6b). Likewise, in this instance, the resonant transition from a C 1s to a π* orbital demonstrated a distinct angular dependence, where the maximum peak π* intensity was observed at the normal incidence angle. Using the same method, we concluded that the ZnPc molecules on the 100 nm thick HAT-CN layer have an azimuthally averaged tilt of 62° relative to the substrate. Despite the error bars in this evaluation, the 5 nm thick HAT-CN leads to a slightly greater *π*–*π* stacking of the ZnPc layer than does the 100 nm thick HAT-CN. However, all ZnPc layers still showed a relatively edge-on orientation, regardless of the orientation of the HAT-CN interlayer.

**Figure 7:**
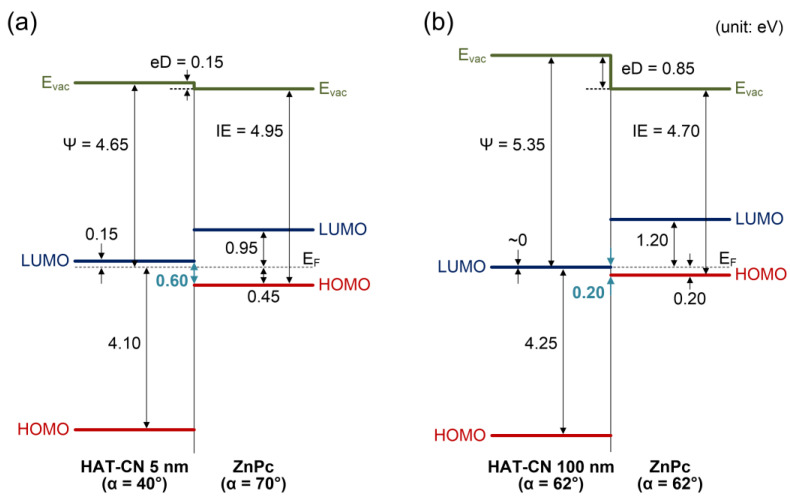
Energy-level diagrams of the (**a**) HAT-CN (5 nm)/ZnPc interface and (**b**) HAT-CN (100 nm)/ZnPc interface. In this diagram, E_vac_ is the vacuum level, eD is the interface dipole, Ψ is the work function, IE is ionization energy, LUMO is lowest unoccupied molecular orbital, HOMO is the highest occupied molecular orbital, and E_F_ is the Fermi level. Units are eV.

Based on the UPS, XPS, and ARXAS results, the energy-level diagrams of the (a) HAT-CN (5 nm)/ZnPc and (b) HAT-CN (100 nm)/ZnPc interfaces are illustrated in Figure 7. The LUMO levels of HAT-CN and ZnPc were estimated by their transport gaps using inverse photoelectron spectroscopy (IPES) [18,56]. The work function and ionization energy of the 5 nm thick HAT-CN layer were 4.65 and 8.75 eV, respectively. The HOMO level of ZnPc was located at 0.45 eV, and the band bending was absent at the interface. The interface dipole was determined using the equation eD = (ΔSEC) − V_b_, where eD is the interface dipole, ΔSEC is the shift in SEC, and V_b_ is the band bending [57]. Charge transfer between the substrate and overlayer is responsible for both interface dipole and band bending, which occur to achieve thermal equilibrium. However, the interface dipole arises from charge transfer within the monolayer region interface, while the band bending arises from charge transfer within a relatively distant region of the interface (~nm). If the charges required for thermal equilibrium are not sufficient within the monolayer region, bend bending occurs. On the other hand, if the charges required for thermal equilibrium are sufficient within the monolayer region, band bending is absent. ΔSEC is evaluated from the substrate to the final thickness (5 nm). For determining the accurate band bending, purely electrostatic effects related to the charge transfer between film and substrate must be considered whereas the final state effects due to differences in polarizability of the medium around the photohole leading to additional binding energy shifts must be eliminated [58]. However, in our case, both HAT-CN and ZnPc organic materials have low free-carrier concentrations and similar dielectric properties. Hence, additional binding energy shifts may not be significant. To evaluate band bending, the energy levels should be determined layer-by-layer. Therefore, we determined the energy level shifts from the 1 nm thick ZnPc layer, which approximately fully covers the HAT-CN layer. The 1 nm thickness also shows a high peak intensity that can determine the onset with sufficient reliability. As a result, we did not observe any band bending in ZnPc layer on both the 5 nm and 100 nm thick HAT-CN layers. An interface dipole of 0.15 eV was observed, and the ionization energy of ZnPc was 4.95 eV. The interface dipole with its negative pole pointing toward the HAT-CN and its positive pole toward the ZnPc decreases the work function of the HAT-CN layer. Therefore, the energy gap between the LUMO level of the HAT-CN and the HOMO level of ZnPc is increased as compared to that in the Schottky-Mott limit (i.e., vacuum level alignment). To improve the charge-injection efficiency based on the charge generation mechanism, it is essential to reduce the energy gap between the LUMO level of the charge-generating HIL (HAT-CN) and the HOMO level of the p-type organic layer (ZnPc). The energy gap between the LUMO level of HAT-CN and the HOMO level of ZnPc is 0.60 eV. By contrast, the work function and ionization energy of the 100 nm thick HAT-CN layer were 5.35 eV and 9.60 eV, respectively. The increasing work function of HAT-CN with increasing thickness can be attributed to a change in the molecular orientation, as indicated by the ARXAS results. While the LUMO level of HAT-CN was very close to the Fermi level (~0 eV), the accurate energy position could not be evaluated because of the large spectral broadening of IPES. The HOMO level of ZnPc was located at 0.20 eV, and the band bending was absent at the interface. An interface dipole of 0.85 eV was observed, whereas the ionization energy of ZnPc decreased slightly to 4.70 eV. In this case, the interface dipole also induces a reduction in the work function of the HAT-CN layer, which consequently leads to an elevation in the energy gap between the LUMO level of the HAT-CN and the HOMO level of ZnPc as compared to the case without the interface dipole. The difference in the ionization energies of ZnPc may originate from the slightly different molecular orientations. Consequently, the energy gap between the LUMO level of HAT-CN and the HOMO level of ZnPc was 0.20 eV. This is a significant decrease compared to that measured for the 5 nm thick HAT-CN sample. This reduction is attributed to the work function, which increased by 0.70 eV, although the magnitude of the interface dipole is larger than that of the HAT-CN (5 nm)/ZnPc case. Therefore, to achieve a high work function via edge-on molecular orientation, which can reduce the energy barrier for hole transport, the HAT-CN HIL must have sufficient thickness.

## 3. Materials and Methods

Experimental methods: for UPS and XPS experiments, an ITO (145 nm thickness and sheet resistance < 15 Ω square^−1^, Thin Film Devices, Anaheim, CA, USA) substrate was cleaned via ultrasonication in deionized water (HPLC grade, Sigma-Aldrich, St. Louis, MO, USA), detergent (Alconox, Sigma-Aldrich, St. Louis, MO, USA), acetone (≥ 99.5%, Sigma-Aldrich, St. Louis, MO, USA), and ethanol (≥ 99.5%, Sigma-Aldrich, St. Louis, MO, USA). The cleaned ITO substrate was then placed in the entry chamber. HAT-CN (≥ 98%, Sigma-Aldrich, St. Louis, MO, USA) and ZnPc (97%, Sigma-Aldrich, St. Louis, MO, USA) were deposited onto the substrate using Knudsen cells via thermal evaporation in the preparation chamber (base pressure: 5 × 10^−7^ Torr). The ITO substrate was at room temperature during deposition. Thickness was monitored using a quartz crystal microbalance (Easy Rate Single Sensor, INFICON, Bad Ragaz, Switzerland). After each deposition step, entirely under vacuum, the sample was transferred to the analysis chamber (base pressure: 1 × 10^−9^ Torr), where UPS and XPS spectra were recorded. To obtain the SEC, we applied −10 V of sample bias. For ARXAS experiments, the ITO substrate was cleaned by Ar^+^ sputtering and annealing under UHV, after which the HAT-CN and ZnPc layers were thermally deposited onto it from well-outgassed thermal evaporators. The ITO substrate was at room temperature during deposition and the film thickness was monitored by a quartz crystal balance. After deposition, the sample was transferred under vacuum into the spectrometer chamber (base pressure: 2 × 10^−10^ Torr) The C K-edge spectra were recorded in the total-electron-yield mode, and the sample drain-current was normalized to the current from a Au-coated mesh positioned in the incident photon beam. The energy scale was calibrated using the first- and second-order diffraction Ti L-edge and O K-edge absorptions of a rutile TiO_2_ reference.

Equipment: For UPS and XPS experiments, a detailed explanation of our in situ analysis system is provided in a previous report [59]. The system comprises a preparation chamber and an analysis chamber connected with a gate valve. An analysis system (PHI 5700, Physical Electronics, Chanhassen, MN, USA) consisting of a spectrometer, a He I_α_ (hν = 21.22 eV) discharge lamp, and an Al K_α_ (hν = 1486.7 eV) X-ray source was employed to record UPS and XPS spectra. The Fermi level of the spectrometer was calibrated by measuring the Au 4f core level and Fermi edge of a clean Au substrate before conducting the main experiment. For ARXAS experiments, ARXAS measurements were performed using the soft X-ray undulator beamline X1B at the National Synchrotron Light Source in Brookhaven National Laboratory (Upton, NY, USA). The X1B beamline is equipped with a spherical grating monochromator, and the photon beam is focused to approximately 60 μm × 40 μm on the sample. The samples were grown in an ultrahigh-vacuum (UHV) organic molecular beam deposition chamber with a base pressure of 2 × 10^−9^ Torr, attached to a multi-technique soft X-ray spectroscopy system.

## 4. Conclusions

In summary, the dependence of the molecular orientation and energy-level alignment of the HAT-CN/ZnPc interface on thickness was elucidated using in situ UPS, XPS, and ARXAS measurements. For the 5 nm thick HAT-CN layer, the HAT-CN molecules were relatively face-on oriented with an azimuthally averaged tilt of 40°, resulting in a work function of 4.65 eV. The HOMO level of the ZnPc layer was 0.45 eV, and the energy gap between the LUMO level of HAT-CN and the HOMO level of ZnPc was 0.60 eV. By contrast, for the 100 nm thick HAT-CN layer, the HAT-CN molecules were relatively edge-on oriented, with an azimuthally averaged tilt of 62°, resulting in a higher work function of 5.35 eV. At this HAT-CN thickness, the HOMO level of the ZnPc layer was 0.20 eV, and the energy gap between the LUMO level of HAT-CN and the HOMO level of ZnPc was reduced to 0.20 eV. This reduction is attributed to the increased work function by 0.70 eV, resulting from the increased thickness, although the magnitude of the interface dipole is larger than that of HAT-CN (5 nm)/ZnPc. This reduced energy gap may improve the hole-transport properties of electronic devices. However, the molecular orientation of the ZnPc layer was not significantly affected by the HAT-CN layer thickness, although the molecular orientation of HAT-CN was changed. These findings highlight the critical role that molecular orientation plays in determining the work function and energy-level alignment of organic HILs necessary for optimizing device performance.

## Data Availability

The data presented in this study are available on request from the corresponding author.

## Sample Availability

Samples of the compounds are not available from the authors.
